# Sensing Free Sulfur Dioxide in Wine

**DOI:** 10.3390/s120810759

**Published:** 2012-08-06

**Authors:** Tanya M. Monro, Rachel L. Moore, Mai-Chi Nguyen, Heike Ebendorff-Heidepriem, George K. Skouroumounis, Gordon M. Elsey, Dennis K. Taylor

**Affiliations:** 1 Institute for Photonics & Advanced Sensing and School of Chemistry & Physics, The University of Adelaide, Adelaide, SA 5005, Australia; E-Mails: tanya.monro@adelaide.edu.au (T.M.); rachel.moore@adelaide.edu.au (R.M.); mai-chi.nguyen@adelaide.edu.au (M.-C.N.); heike.ebendorff@adelaide.edu.au (H.E.-H.); 2 School of Agriculture, Food and Wine, The University of Adelaide, Waite Campus, PMB 1, Glen Osmond, SA 5064, Australia; E-Mails: george.skouroumounis@adelaide.edu.au (G.K.S.); elsey_gm@hotmail.com (G.M.E.)

**Keywords:** sulfur dioxide, potassium (sodium) metabisulfite, wine, microstructured optical fiber, sensors, wine sensing, pararosaniline

## Abstract

Sulfur dioxide (SO_2_) is important in the winemaking process as it aids in preventing microbial growth and the oxidation of wine. These processes and others consume the SO_2_ over time, resulting in wines with little SO_2_ protection. Furthermore, SO_2_ and sulfiting agents are known to be allergens to many individuals and for that reason their levels need to be monitored and regulated in final wine products. Many of the current techniques for monitoring SO_2_ in wine require the SO_2_ to be separated from the wine prior to analysis. This investigation demonstrates a technique capable of measuring free sulfite concentrations in low volume liquid samples in white wine. This approach adapts a known colorimetric reaction to a suspended core optical fiber sensing platform, and exploits the interaction between guided light located within the fiber voids and a mixture of the wine sample and a colorimetric analyte. We have shown that this technique enables measurements to be made without dilution of the wine samples, thus paving the way towards real time *in situ* wine monitoring.

## 1. Introduction

In the wine industry, sulfur dioxide (SO_2_) is frequently added to must and juice as a preservative to prevent bacterial growth and slow down the process of oxidation by inhibiting oxidative enzymes. SO_2_ also improves the taste and retains the wine's fruity flavors and freshness of aroma [1]. It is commonly added as either potassium or sodium metabisulfite which, upon addition, forms a pH dependent speciation in solution.

At low pH, the predominant species is molecular sulfur dioxide (SO_2_), which exhibits germicidal properties. However, at the pH of wine (between 3.0 and 3.8) the major species is the bisulfite anion (HSO_3_^−^), which acts as an antioxidant [2]. In this work the collective term used for all species of SO_2_ found in wine is ‘sulfites’.

Two classes of sulfites are found in wine: free and bound. The free sulfites are those available to react and thus exhibit both germicidal and antioxidant properties. The bound sulfites are those that have reacted (both reversibly and irreversibly) with other molecules within the wine medium. The sum of the free and bound sulfites defines the total sulfite concentration.

Most countries have strict guidelines as to the maximum levels of total sulfites permissible in wine. From a winemaking point of view, high concentrations of sulfites can affect the sensory attributes or characteristics of the wine. Additionally, too much sulfur in the must also delays the malolactic fermentation of wine, particularly in wines with low pH [1]. For these reasons, the concentration of sulfites in wine must be closely monitored and regulated.

There are two internationally recognized methods for the quantification of free and total sulfites in wine or must [3]. The traditional method is the Monier-Williams method [4], where the sample of wine is acidified and aspirated into a solution containing hydrogen peroxide, which forms sulfuric acid that is then titrated against a sodium hydroxide solution where a mixed indicator is used to determine the ‘end point’. Several variations of the Monier-Williams method exist for wine analysis including the Modified Monier Williams method [5], the Optimized Monier-Williams method [6] and the aspiration (aeration-oxidation) method [7]. The latter is most often employed for sulfite determination of wine and must in Australia [7]. The aspiration method gives reproducible results and is accurate up to 5% compared to the Monier-Williams method for both red and white wines [8]. However, this method has several drawbacks including the requirement for use of a relatively large sample volume (20 mL for each sample) and the 15 min reaction time, and is also sensitive and thus susceptible to experimental error. The second method for the analysis of free and total sulfites in wine is the Ripper method [9] utilizing a direct titration of the wine sample with iodine using a starch indicator. This method is less accurate than the Monier-Williams method and has several drawbacks [8]. This method is suitable for use as a quick method estimating the sulfite concentration in wine [10].

Other techniques have also been developed to detect sulfites in wines. These include colorimetric [11–13], electrochemical [14,15], chemiluminescence [16], chromatrographic [17], fluorometric [18] and enzymatic [19] methods. Many of these methods are implemented using flow injection systems that incorporate a gas diffusion unit to remove the molecular SO_2_ from the liquid prior to detection.

One of the colorimetric techniques that have been used to detect free sulfites utilizes pararosaniline hydrochloride (PRA), which is a highly conjugated amine salt and one of the components of the dye ‘basic fuschin’. The addition of hydrochloric acid and formaldehyde to aqueous PRA produces a solution, light purple in color, which turns deep violet when exposed to sulfites. This reagent can be used as a quantitative test for sulfites based on absorption spectroscopy [20].

**Figure 1.**
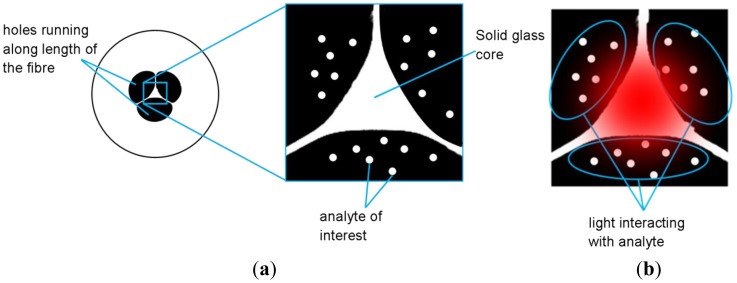
(**a**) Cross section of the suspended core optical fiber used in this wine sensing platform; (**b**) Red light being launched into the fiber; some of this light overlaps with the analyte and is absorbed.

Microstructured optical fibers (MOFs) have an array of micron-scale air holes that run along the length of the fiber [21]. These fibers can be designed such that a significant fraction of the light that is guided along the fiber is located within the holes, where it is then available to interact with gases or liquids that are loaded into these holes (Figure 1) [22]. One variant of microstructured fiber that has been extensively used for sensing is the suspended-core optical fiber, where the core is solid glass [23]. The interaction of the light with the species filling the fiber air holes allows the fibers to be used for sensing techniques such as absorption [22,24] and fluorescence-based sensing [23,25]. These fibers can serve as platforms that allow for sensitive, low volume sensing.

Here, for the first time, we demonstrate a novel dip sensing platform for wine based on a colorimetric technique deployed within a microstructured optical fiber. This technique enables the quantification of free sulfites in small volume samples, including liquid model wine solutions and several finished white wine samples. This technique utilizes colorimetric reaction between pararosaniline (PRA), formaldehyde and sulfites [20]. This reaction was first characterized in cuvettes employing a UV−Vis spectrophotometer, and was then incorporated into a platform based on suspended core optical fibers to enable sensitive and low volume absorption analysis of wine samples.

## 2. Experimental Section

### 2.1. Chemical Reagents

All chemicals were of analytical reagent grade with no further purification. Millipore water was used throughout the experiments. The PRA solutions and model wine solutions were made fresh on each day of analysis.

### 2.2. Pararosaniline Solutions

A concentrated pararosaniline stock solution (3.088 × 10^−3^ mol/L) was made by dissolving pararosaniline hydrochloride (Sigma Aldrich, Sydney, Australia) in a 10% aqueous ethanol solution. The concentrated stock solution was used to make PRA working solutions. Working solutions contained pararosaniline hydrochloride, hydrochloric acid (HCl) and formaldehyde in the molar ratio of 1:1255:125 respectively [11]. For example, a working solution with PRA concentration of 4.68 × 10^−4^ mol/L in 25 mL contained 3,790 μL of concentrated PRA stock (3.088 × 10^−3^ mol/L), 1,439 μL of 32% HCl and 189 μL of 36% formaldehyde solution; the volume was then completed to 25 mL with water. The concentration of PRA in the working solution was adjusted to ensure that enough PRA molecules were present in solution to react with all of the sulfite molecules.

### 2.3. Model Wine

Model wine solutions were made via dilution from a stock model wine. The stock model wine solution contained sodium metabisulfite dissolved in 10% ethanol in saturated potassium bitartrate (>5.7 g/L in water) solution. The purity of the sodium metabisulfite had previously been determined (via the aspiration method) to be 92% and the final concentration of sulfites in the stock was adjusted accordingly. The required volume of model wine stock solution was pipetted into volumetric flasks and topped with 10% ethanol in saturated potassium bitartrate solution to produce model wine solutions in the range of 1–100 ppm of sulfites.

### 2.4. Wine Samples

Two white wines were sourced from local supermarkets; Jacob's Creek Sauvignon Blanc (12% alcohol) and Hardys Pinot Grigio (12% alcohol). The wine samples were stored at room temperature and opened on the day that they were used.

### 2.5. Fiber

**Figure 2.**
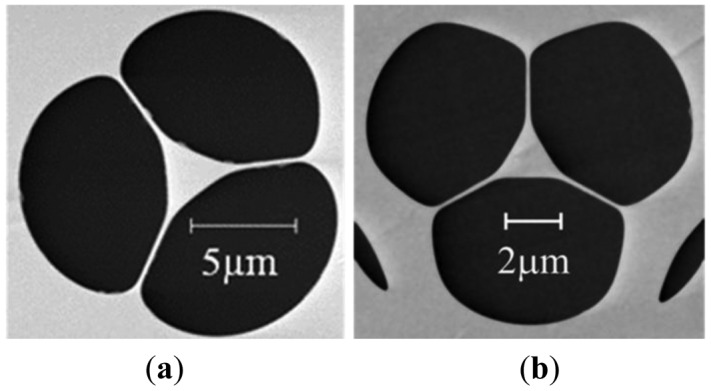
Scanning electron micrograph (SEM) of the cores of the fabricated F2 glass suspended core optical fibers used for this study. (**a**) Fiber 1, core diameter 2.1 μm; (**b**) Fiber 2, core diameter 1.39 μm.

The suspended core optical fibers used for this study was fabricated in-house from commercially sourced lead silicate glass (Schott F2 glass) using the billet extrusion and fiber drawing technique [26]. Two different fibers with different core sizes were utilized. Figure 2 shows a scanning electron micrograph (SEM) of the core of the fabricated fibers. The first fiber had an outer diameter of 160 μm and a core diameter of 2.1 μm, Figure 2(a), and the second fiber, Figure 2(b), had an outer diameter of 130 μm and a core diameter of 1.4 μm. Smaller cores enable access to larger light-liquid overlap, and thus higher sensitivity in principle, whilst larger cores offer more straightforward optical coupling and lower loss. The standard absorption of these fibers at 532 nm is approximately 1.12 ± 0.1 dB/m.

### 2.6. In-Cuvette Measurements

A Varian Cary 500 spectrophotometer (Varian Australia, Melbourne, Australia) was used for all in-cuvette absorbance measurements. Measurements utilized double beam mode at full slit height in the UV–Vis mode through plastic cuvettes (4 mL internal volume) with a 1 cm path length. Varian UV Scan application software version 3.00 (399) was used for single time point data acquisition. Varian scanning kinetics software was used when absorbance was measured as a function of time. Water was used as the baseline correction, and a heated cell block was used to control the temperature during analysis.

Both single point and kinetic analyses were performed. For the single time point absorption analysis, equal volumes of PRA working solution and model wine samples were pipetted into a cuvette and completed to 3 mL with water and left to develop for 10 minutes. When performing kinetic experiments, equal volumes of the solutions were mixed directly in-cuvette and diluted to 3 mL. A small magnetic stirrer was used to ensure adequate mixing of the sample during analysis. The cuvette was sealed with tape to avoid evaporation or potential loss of SO_2_ gas and assist with obtaining reproducible results.

The dilution factor used was selected depending on the concentration of the PRA working solution and model wine solutions to ensure that the absorption values obtained were within the optimal range of the spectrometer. Model wine samples were analyzed without replication, however, white wine samples were analyzed in triplicate as a means of testing the reproducibility of this novel sensing platform.

### 2.7. In-Fiber Measurements

**Figure 3.**
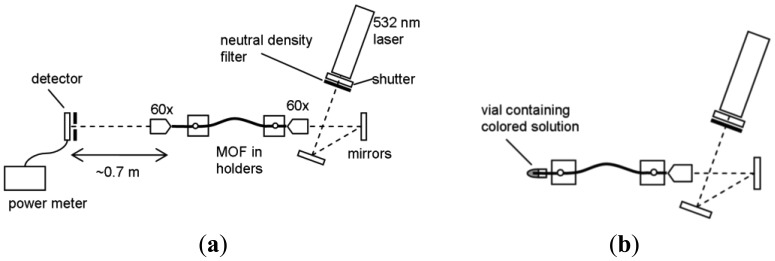
(**a**) Experimental set up for the in-fiber absorption measurement (not to scale). (**b**) Schematic of the filling phase of the absorption measurement.

The schematic of the in-fiber experimental set up is shown in Figure 3(a). The two ends of the fiber were placed in fiber holders on three-axis nano-translation stages for accurate alignment of the light into and out of the fiber. Light from the 25 mW 532 nm laser (CrystaLaser, Reno, NV, USA) was attenuated using a neutral density filter (ND 2) and launched into the core of the fiber using a 60× objective lens. At the other end of the fiber, a pinhole was used to ensure that any light guided within the cladding of optical fiber (rather than the core) is not incident on the detector. The transmitted light guided by the core was focused at the pinhole plane using a 60× objective lens. The pinhole was set to let the light from the fiber core to pass through to the detector, which was connected to a power meter.

The solutions used in the cuvette experiments were also used for the in-fiber measurements. Equal volumes of the PRA stock solution and the model wine solution were pipetted into a vial and left for at least 10 minutes to allow the color to develop. It should be noted that for the in-fiber measurements, no further dilution was found to be necessary. This is one advantage of the optical fiber sensing platform; by choosing the size of the core of the optical fiber appropriately, it is possible to reduce the light-liquid interactions to the point where concentrated samples can be analyzed without dilution.

For each absorbance measurement, a piece of optical fiber was cleaved and put on the fiber holders and held in place with magnets. Approximately 2 cm of the fiber on the filling end was left to protrude from the edge of the fiber holder (Figure 3(b)). The laser input translation stage was adjusted to optimize the launch of the laser light into the core of the fiber. The coupling was then maximized by focusing the transmitted light at the pinhole plane and launched onto the detector and adjusting the translation stage on the laser end to maximize the optical power. The power was recorded over a 30 second period. The microscope objective at the filling end was then removed and the vial containing the mixed solution was put in a holder to allow the fiber tip to be immersed in the liquid whilst maintaining the alignment of the laser light into the fiber. The fiber was left to fill via capillary forces for a set time before the vial was taken away. The objective was then replaced on the filling end, and refocused on the pinhole plane with the laser as before. The power was recorded over a 30 second period. Immediately after this measurement, the filled length of the fiber was measured under a microscope. A new length of fiber was used for each measurement.

The fiber lengths and filling times were different for the two different fibers used as a result of differences between the sizes of the holes in each fiber, and this information is summarized in Table 1. The differences in length were taken into account by calculating the absorbance per cm of filled fiber. Initial results obtained with fiber 1 produced results with a lot of scatter and therefore in an effort to reduce the spread in the data, subsequent solutions analyzed with fiber 2 were first filtered through a 0.45 μm pore filter prior to filling the fiber to ensure that any particulates present did not interfere with the measurements.

## 3. Results and Discussion

### 3.1. Preliminary In-Cuvette Optimization

Prior to performing any in-fiber measurements, preliminary experiments were performed in-cuvette to optimize the experimental technique using model wine.

### 3.2. Reaction Mechanism

**Figure 4.**
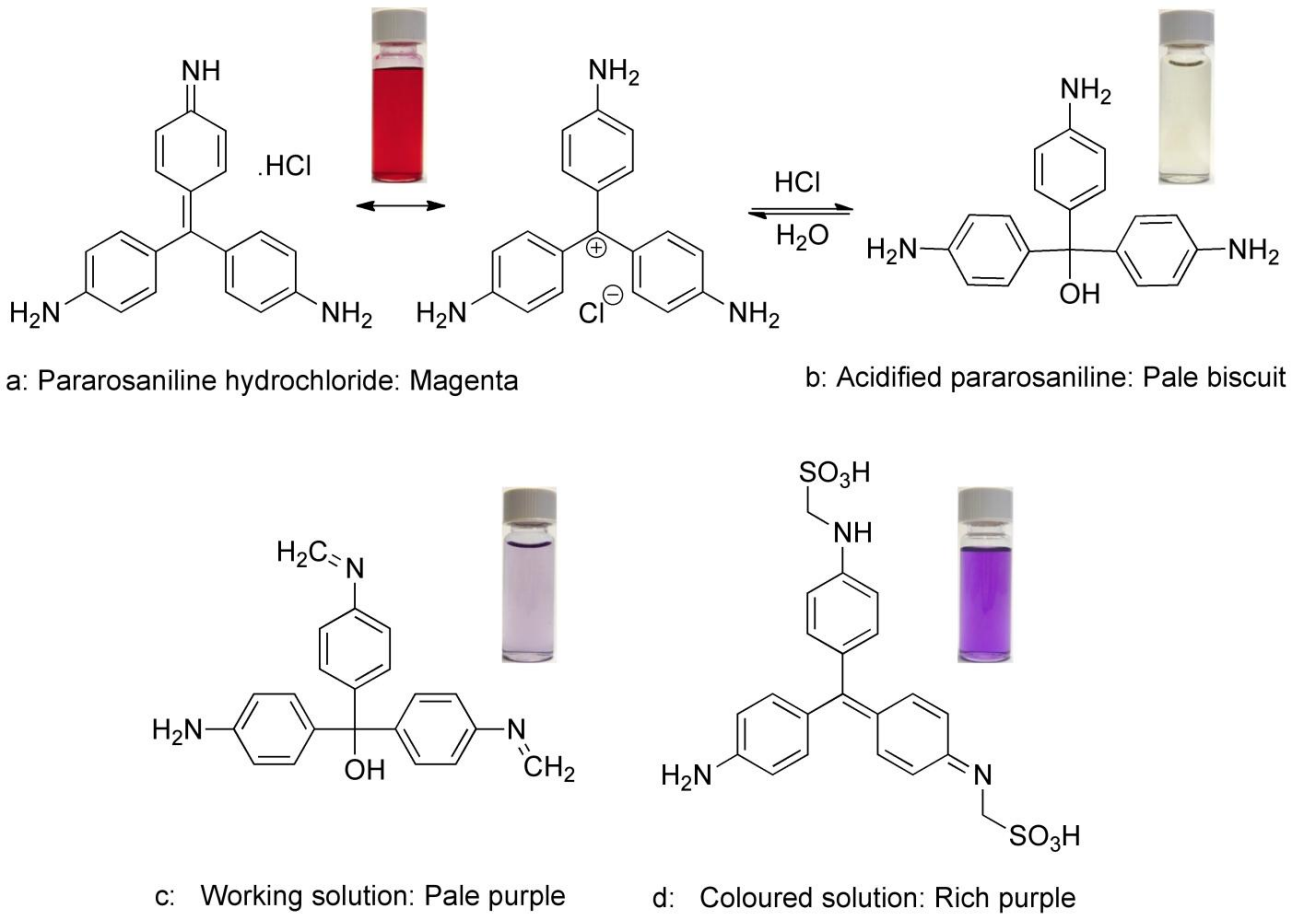
Chemical structures of the four compounds produced throughout the pararosaniline reaction and its corresponding colors. (**a**) Pararosaniline hydrochloride; (**b**) Acidified pararosaniline; (**c**) Pararosaniline working solution; (**d**) Sulfonic acid mixture with model wine with 30 ppm sulfite solution in a 1:1 ratio.

Pararosaniline hydrochloride forms a bright magenta solution (Figure 4(a)) when dissolved in aqueous solution. Upon acidification with hydrochloric acid, the solution is essentially bleached due to diminished conjugation resulting in a pale biscuit colored solution (Figure 4(b)). The addition of formaldehyde results in initial formation of the iminium ion and affords a pale purple solution (working solution) (Figure 4(c)), which reacts extremely readily with sulfites to ultimately form the highly conjugated alkyl amino sulfonic acid (colored solution) (Figure 4(d)) as a rich purple solution which has a peak absorbance between 550 and 560 nm.

The stoichiometry of this reaction between PRA to sulfites is not well understood. It has been suggested that when PRA is present in large excess, that the mono-substituted product is formed [27], however Huitt and Lodge [28] claim that the spectrally active product is an equilibrium mixture of mono-, di-, and tri-substituted pararosaniline and hence it was important to identify the effect of PRA concentration on the absorbance values of the final product, as well as the time required to achieve the optimal absorbance.

### 3.3. Stoichiometry

**Figure 5.**
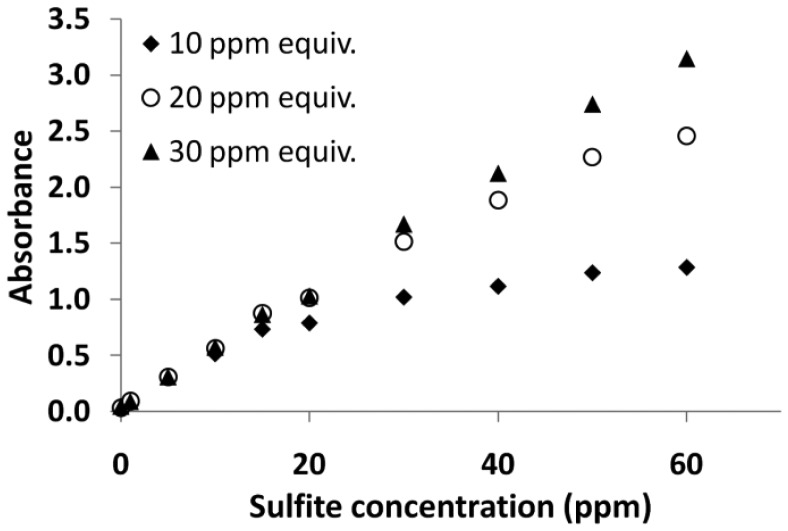
Maximum absorbance reading for three concentrations of working solution as a function of model wine sulfite concentration.

In order to investigate the level of substitution and its impact on the maximum absorbance obtained, three PRA working solutions were produced with PRA concentrations of 1.56 × 10^−4^, 3.12 × 10^−4^ and 4.68 × 10^−4^ mol/L. These concentrations are equivalent to sulfite concentration of 10, 20 and 30 ppm (1:1 ratio). The latter sulfite concentration is typical of a finished white wine whilst the former would be considered to be a level where the wine is highly susceptible to oxidation or spoilage. Each of the PRA solutions (300 μL) was mixed with model wine solutions (300 μL) containing 0–60 ppm of sulfites and diluted with water. Absorbance was measured over a 400 nm wavelength range to identify the position of the maximum absorbance obtained and also to investigate peak shape. Figure 5 illustrates the highest absorbance value obtained for each resultant colored solution plotted against sulfite concentration. This clearly illustrates the importance of the stoichiometry for this reaction. The graph illustrates the point where there are insufficient PRA molecules available to react with all of the sulfites in solution and hence the development of color, and therefore absorbance, begins to plateau. It is interesting to note that until this point, the absorbance values of the three PRA colored solutions were similar, suggesting that the maximum absorbance values obtained are independent of the PRA concentration provided that enough PRA exists to react with the sulfites. Figure 5 shows that the absorbance values of the lowest concentration of PRA (1.56 × 10^−4^ mol/L) begins to deviate from the trend between 10 and 15 ppm of sulfites, and by 20 ppm the plateau effect is more evident, a similar observation can be made for the mid-strength PRA solution at a concentration between 30–40 ppm sulfite concentration. The most concentrated PRA working solution was able to maintain linearity in absorbance until 60 ppm, suggesting that the di-substituted alkyl amino sulfonic acid predominates. From this point forward, a 1:2 molar ratio of PRA:sulfite was used to find the appropriate concentration in the working solution for the detection of sulfites in wine.

### 3.4. Color Development

**Figure 6.**
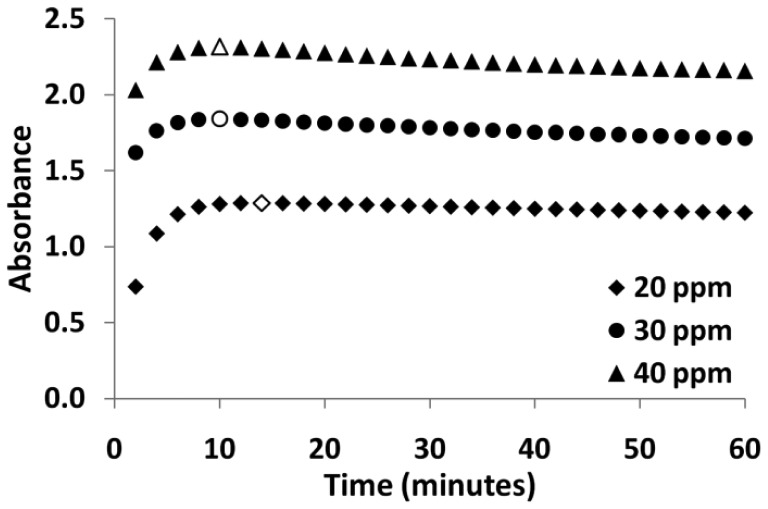
Maximum absorbance values obtained as a function of time for three concentrations of sulfites in solution. Hollow data points illustrate the maximum absorbance value obtained.

The color development of the final solution was analyzed as a function of time. In order to investigate the color development, equal volumes of working solution (300 μL of 6.24 × 10^−4^ mol/L PRA) and model wine samples were combined, diluted to a final volume of 3 mL and allowed to develop in-cuvette using a small magnetic stirrer for mixing. Absorbance measurements were taken at 2 minutes intervals, and the highest absorbance value obtained at each time point were plotted against the development time for 3 concentrations of model wine solutions (Figure 6). This graph illustrates that color development is essentially complete by 10 minutes for these solutions. Consequently, a development time of 10 minutes was used.

### 3.5. Wavelength Dependence

Initial analysis revealed that the concentration of PRA in the working solution can alter the shape of the spectra at wavelengths larger than the maximum (greater than ∼550 nm). However, at wavelengths shorter than the maximum, large differences were not observed. In order to observe the maximum peak height and to establish a calibration curve, a concentrated PRA working solution (150 μL of 7.80 × 10^−4^ mol/L) was mixed with model wine solutions (150 μL) ranging in sulfite concentration from 1–100 ppm and diluted to a final volume of 3 mL. The colored solution was developed for 10 minutes in-cuvette and the absorbance was recorded from 700–350 nm. The spectra revealed that the maximum absorbance occurred at approximately 550 nm. However, in order to use this colorimetric technique within the suspended core optical fiber platform, it was important to ensure that a linear calibration curve could be achieved at the available laser wavelength, which in this case was 532 nm.

**Figure 7.**
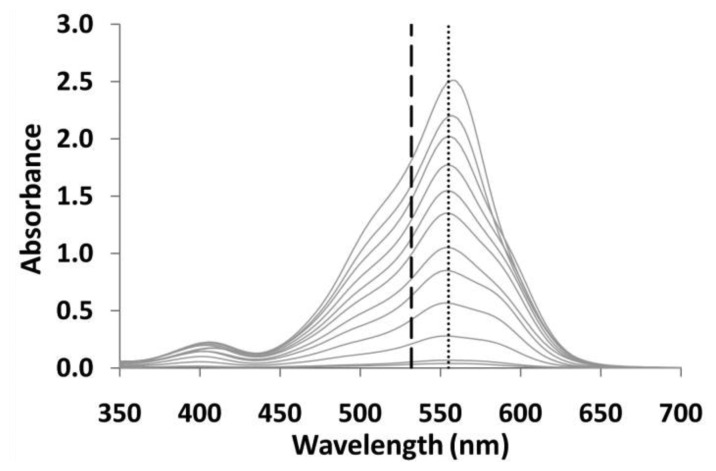
Full absorption spectra of colored solution with 0–100 ppm of model wine solution. Vertical lines illustrate the maximum absorbance obtained, and the absorbance at 532 nm.

**Figure 8.**
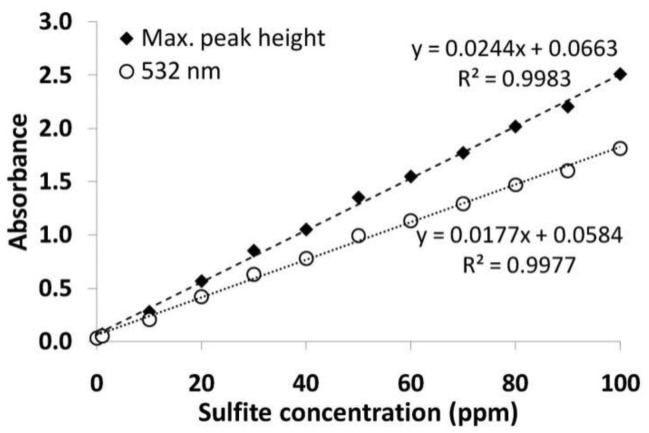
Calibration curves obtained at the maximum absorbance and at 532 nm for solutions containing 0–100 ppm of sulfites using a 7.8 × 10^−4^ mol/L working solution.

Figure 7 illustrates the full absorbance spectra of each of the colored solutions (0, 1, 10, 20, 30 → 100 ppm of sulfites). The resulting calibration curves for the maximum absorbance and the absorbance at 532 nm can be seen in Figure 8. These calibration curves show that the absorbance is linear, at both positions of the spectra (with an R^2^ of 0.9977 at 532 nm and 0.9983 at the maximum peak) and hence the results obtained in cuvette were suitable for use within the fiber sensing platform.

These experiments demonstrate that a linear calibration curve exists at several wavelengths shorter than the maximum peak height and additionally, these calibration curves will still be valid for several concentrations of PRA working solution, provided that that concentration of sulfites does not exceed the concentration of PRA by more than 2:1.

### 3.6. Preliminary Fiber Work

Based on the cuvette absorption measurements and the methodology developed above, this approach was then adapted for use with the convenient optical fiber sensing platform.

The absorbance of the mixture in the fiber, *A*, was calculated using:

(1) $$A=\left[\log_{10}\left(P_{\text{unfilled}}/P_{\text{filled}}\right)\right]/L$$

where *P_unfilled_* is the transmitted power of the unfilled fiber, *P_filled_* is the transmitted power when it is filled with the mixture of colored solution, and *L* is path length (the filled length of the fiber). According to the Beer-Lambert law, the absorbance is defined as *A = εLcPF*, where *ε* is the molar absorptivity of the absorbing material, *L* is the path length, *c* is the concentration of the sample and *PF* is the power fraction. The power fraction is the fraction of the guided light within the fiber that is available to interact with the absorbing material (*i.e.*, the fraction of the guided light that is located within the liquid-filled holes).

For cuvette measurements, all the light interacts with the sample, so the power fraction can be considered to be equal to 1.0. However, for a MOF with a core of >1 micron in diameter, for visible wavelengths most of the light is confined within the core with only a small fraction spreading into the holes of the fiber, allowing interaction with the solution that fills these holes. The power fraction is dependent on wavelength, material type and fiber structure [29]. As mentioned previously, fibers with a relatively large core diameter have a smaller power fraction compared to fibers with a smaller core diameter. Thus, to determine if the cuvette measurements agree with the fiber measurements, one can keep all parameters constant (same samples, path length, sulfur dioxide concentration) and compare the fiber's theoretical power fraction with the calculated power fraction by estimating the ratio of the slopes of the two calibration curves). The power fraction for fiber 1 was calculated to be 1.8 × 10^−3^ and the power fraction for the smaller core fiber 2 was calculated to be 8 × 10^−3^. These calculated were performed analytically by approximating the MOFs by an air-suspended rod surrounded by water and excited with light at 532 nm.

**Figure 9.**
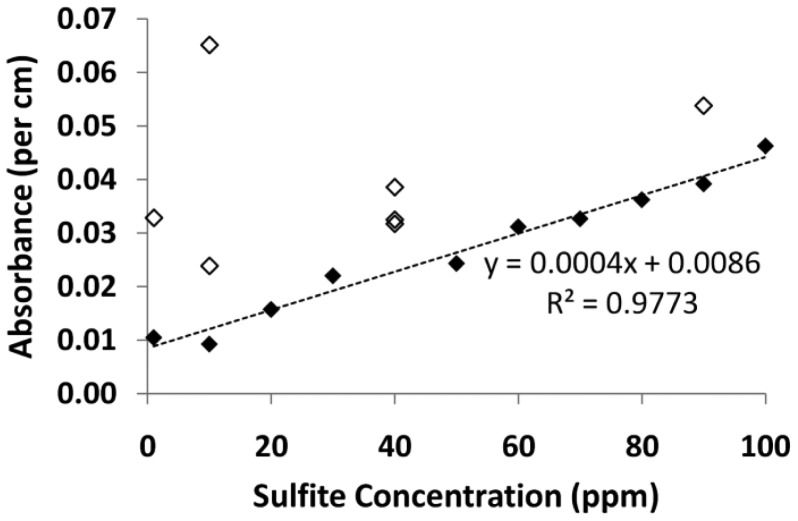
Calibration curve from 1–100 ppm using 7.8 × 10^−4^ mol/L PRA working solution in fiber 1. Data points not included in the calibration curve are shown as the hollow data points.

The calibration curve for fiber 1 was plotted and is depicted in Figure 9. Some of the absorbance readings were higher than expected, possibly due to particulates settling on or near the fiber core and giving incorrect (high) readings, these results are illustrated (hollow data points), although they were not used to determine the line of best fit. Based on this, the solutions in subsequent measurements were filtered prior to filling the fiber. For the remaining data points in the range of sulfite solutions tested, good linearity was demonstrated (R^2^ = 0.9773).

**Figure 10.**
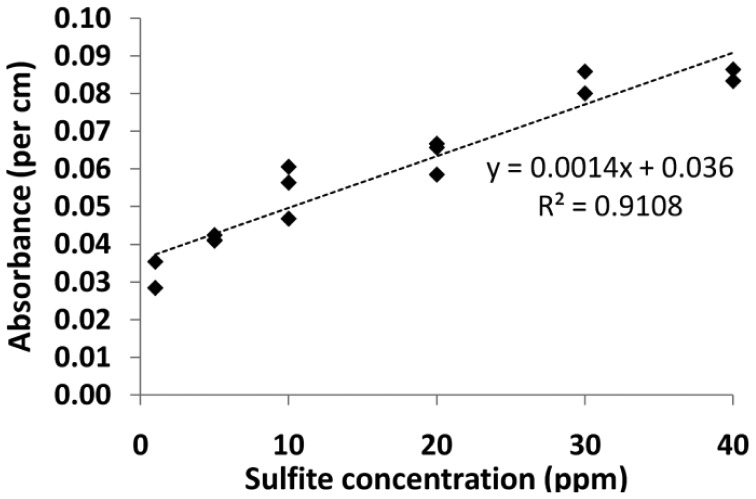
Calibration curve from 1–40 ppm using 6.24 × 10^−4^ mol/L PRA working solution in fiber 2.

The calibration curve for fiber 2 is shown in Figure 10. This data is much less scattered due to the filtering of the sample (consistent with the hypothesis that in the previous results, large particulates may have adhered to the core), and the R^2^ value of the calibration curve is 0.9108. As the core of fiber 2 is smaller than fiber 1, the interaction between the sample and the light is higher and thus the calibration curve has a higher slope.

To allow direct comparison between the calibration curves of the cuvette and in-fiber measurements, the slopes of the calibration curve were corrected to account for the different dilutions used for the two methods. The sulfite concentration was diluted by a factor of 2 for the fibers (by mixing equal volumes of model wine and PRA working solutions), and by a factor of 20 for the cuvette measurements (to ensure that the absorbance measurements were within the working range of the spectrometer), thus the slopes were multiplied by 2 and 20 respectively. Table 2 summarizes the comparison of the theoretical and calculated power fraction. For the measurements with fiber 2 where sample filtering was used, the measured power fraction was close to the theoretical power fraction (showing just a 1% difference). This shows that the results achieved in cuvette can be translated to the fiber platform.

### 3.7. White Wine Samples

**Figure 11.**
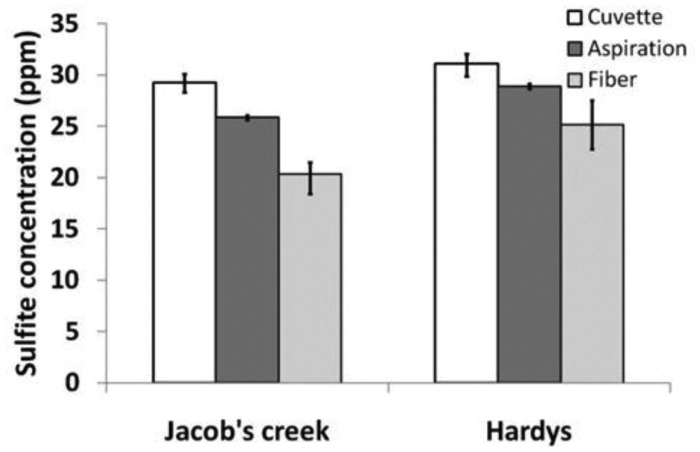
Sulfite concentration in two white wine samples utilizing three quantification methods. Graphical representation of the average results and error bars represent the maximum and minimum results obtained.

The sulfite concentration for two different white wines was analyzed and quantified using three different techniques; the aspiration method (aeration-oxidation), PRA method in cuvette and the PRA method in MOF. The average results for the three methods are illustrated in Figure 11.

The aspiration analysis was performed as outlined by Iland *et al.* [10] however the suggested concentration of the sodium hydroxide solution was diluted 10 fold in an attempt to achieve greater accuracy in the titration results. Each wine was tested in duplicate.

The PRA analysis performed in cuvette utilized a 7.80 × 10^−4^ mol/L working solution which was mixed in equal amount with the white wine sample (150 μL), diluted, developed and analyzed in triplicate. The absorbance measurements at the maximum peak height were used in conjunction with the calibration curve illustrated in Figure 8 to determine the concentration of free sulfites in the wine.

The PRA method in MOF utilized a 7.80 × 10^−4^ mol/L, which was mixed in a 1:1 ratio with the white wine sample and analyzed in quadruplicate using fiber 2. The absorbance measurements were made according to the method outlined previously. Development times for each of the samples ranged from 17 minutes to 80 minutes. The calibration curve for fiber 2 (Figure 10) was then used to determine the sulfite concentration in the wine.

The quantitative comparison of the three analysis techniques presented here for sensing free sulfur dioxide in wine is promising. The results for each of the methods illustrate the same trend; with the Hardys wine containing a higher concentration of sulfites. It is possible that absorbance spectra of the wine influenced the absorbance of the colored solution, and if this were to be substantiated this effect could be removed by changing the sensor design, for example by using a different laser wavelength.

## 4. Conclusions

The results reported here indicate that the detection of sulfites in model wine with suspended core optical fibers has the potential to be developed into a platform that can achieve constant, near real-time wine monitoring cheaply and autonomously. By tailoring the core size of the fiber, the sensitivity of the system can be modified such that the detection of the sulfites can be carried out without dilution of the solution. The fibers can also be bundled and utilized to do the same analysis at the same time point. This will enable an accurate average readout of the sulfite level in the wine with minimal volume loss, an excellent improvement on current laboratory procedures. The non-intrusive system reported here will also reduce the risk of spoilage, as a result of oxygen being introduced into the barrel headspace when large volumes of wine are removed for analysis and causing substantial ullage. Sulfite measurement is considered the most important measurement in wine analysis, and guides the winemaker on the wine's protected state, *i.e.*, microbial spoilage and potential oxidative destruction. When considering barrel storage areas, containing thousands of barrels, only a randomly selected set of barrels is usually analyzed by the current techniques due to the sheer number of barrels and the time and cost of analysis. MOF technology will enable all barrels to be analyzed, not a selected subset, and at a fraction of the time. Furthermore, the MOF technology developed herein has the ability to be developed for different analyses, such as acidity, temperature, sugar content and microorganism or wine taint detection, all of which could be bundled together. It is this attraction that will become the forefront of modern chemical analysis of wines in the near future after further development. It is the simplicity of this technique that may enable it to be utilized on a large scale and allow winemakers to ensure the health of their wines throughout the wine making process, not just wines in barrel storage and at minimal cost. Clearly there are many potential applications including, but not limited to, an early warning device that could alert the winemaker when levels of sulfites are outside of the desired range.

## Figures and Tables

**Table 1.** Parameters utilized when using fibers 1 and 2.

**Fiber Number**	**Fiber length (cm)**	**Filling time (minutes)**	**PRA concentration in working solution (mol/L)**	**Sample filtering**
**1**	19 ± 0.2	7	7.80 × 10^−4^	No
**2**	16 ± 0.2	5	6.24 × 10^−4^	Yes

**Table 2.** Comparison of the fiber power fraction and ratio of the slopes. The slope of the cuvette calibration curve (0.0177) is taken from Figure 8 at 532 nm.

	**Corrected calibration curve slope**	**Slope_fiber_/Slope_cuvette_**	**Theoretical power fraction**	**% Difference**
Fiber 1	0.0008	2.3 × 10^−3^	1.8 × 10^−3^	+26
Fiber 2	0.0028	7.9 × 10^−3^	8 × 10^−3^	−1
